# High Tuberculosis and HIV Coinfection Rate, Johannesburg

**DOI:** 10.3201/eid1305.060908

**Published:** 2007-05

**Authors:** Melanie-Anne John, Colin Nigel Menezes, Gajendra Chita, Ian Sanne, Martin Peter Grobusch

**Affiliations:** * National Health Laboratory Services, Johannesburg, South Africa; † University of the Witwatersrand, Johannesburg, South Africa; ‡ Helen Joseph Hospital, Johannesburg, South Africa

**Keywords:** Human immunodeficiency virus, tuberculosis, coinfection, South Africa, letter

**To the Editor:** Tuberculosis (TB) is the leading cause of illness and death among HIV-1–infected patients in sub-Saharan Africa (*1*–*3*), but valid data on the population-level interaction between the TB and HIV epidemics are scarce (*4*). Our objective was to determine the extent of this dual epidemic in our setting, a hospital in Johannesburg, South Africa. We did this by introducing bedside TB and HIV counseling. We also intended to increase the use of voluntary counseling and testing for our TB patients and facilitate referral to our antiretroviral clinic.

From February to April 2006, 2 volunteers from Community AIDS Response (CARE) counseled patients admitted to the medical wards of the Helen Joseph Hospital. This regional hospital serves a catchment population of >500,000 people, predominantly low-income black Africans. Counselors provided TB and HIV wellness and adherence information, HIV pretest counseling, and referral to the Themba Lethu Clinic for rapid testing that used standard CARE modules.

Basic demographic, TB, and HIV data from patient records were documented on standard data collection forms. Missing data were extracted from the hospital database and Therapy Edge-HIV, the data management system used by the HIV clinic. HIV testing was conducted with a fourth-generation ELISA or rapid finger prick antibody test, according to World Health Organization guidelines.

Most admissions were for pulmonary TB. A total of 467 patients receiving TB treatment were counseled; 8 of these patients refused the TB counseling service, and 2 refused voluntary counseling and testing for HIV. These 467 patients constituted 13% of medical admissions and excluded the 1,075 patients seen at the hospital’s outpatient clinic with suspected TB for this 3-month period. Our impression is that this figure constitutes an underrepresentation of the total TB admissions because TB counselors were not able to see every patient with TB.

Laboratory data were retrievable for 373 inpatients. For 301 (81%) of the 373 patients, TB blood culture, smear, or culture results could be traced. Hence, 72 (19%) of 373 patients who were receiving TB treatment had no record of a diagnostic effort to confirm TB. A total of 284 (76%) HIV test results could be traced; 270 (95%) of the 284 accessible TB patients had concurrent HIV infection (Table).

**Table:** Method of HIV diagnosis, Johannesburg, South Africa

Method	No. positive (%)	No. negative (%)
ELISA	110/123 (89)	13/123 (10)
Rapid test	32 (100)	32/32 (0)
Clinical diagnosis only	61 (100)	61/61 (0)
HIV status known to patient	67/68 (98)	1 (1)
Total	270/284 (95)	14/284 (5)

Most (123 [89%]) documented HIV results were from ELISAs performed during admission. Rapid testing performed in the ward was unacceptable to patients because confidentiality was compromised in large, busy wards and patients were often too ill to move to a side room. The system of making an appointment with the HIV clinic at the time of discharge failed because few patients (5%) actually had the rapid test after admission or began antiretroviral therapy. Those who began such therapy would have been captured on our database.

The level of concurrent TB and HIV coinfection at the hospital was 95%. To the best of our knowledge, this is the highest level ever described in the peer-reviewed English-language literature (*5*). This finding may reflect the selection bias for our inpatients, who generally would have more coexisting conditions than outpatients do. Also, HIV data were missing for 24% of the 373 patients, a fact that may also influence this finding.

The peak age incidence of TB in our population corresponds with previously published data and is similar to the peak age incidence of the HIV epidemic in South Africa (*6*). In one third of the admitted patients, no TB investigations were undertaken. This may be because patients provided a history of TB diagnosed elsewhere, or it may reflect the high rate of sputum smear negativity in the HIV-infected population, which lowers the clinician’s threshold for empiric TB treatment.

Mycobacteremia appeared to be less common (14%) than reported in other African studies (*7*). However, we did not have a complete dataset—only 195 (52%) of the 373 patients could be evaluated.

TB and HIV have reached unprecedented levels in our urban inpatient population. TB and HIV must be viewed as different sides of the same coin, and services and staff must change accordingly. We need to use the opportunity of hospital admission to educate patients on the interaction between these 2 epidemics and facilitate patient referral for long-term management. Such management would include voluntary counseling and testing, as well as antiretroviral medication. The latter is a recognized strategy of TB control because it reduces the risk for TB by 70%–90% (*8*).

In addition, all inpatient procedures in our TB/HIV control programs need to be strengthened. Infection control interventions to limit the high rates of nosocomial transmission of TB to other vulnerable patients and staff need to be instituted. At our hospital, we are committed to these approaches. To this end, we have secured a Presidents Emergency Plan for AIDS Relief Grant via the nongovernmental organization Right to Care, which shares our vision. Urgent and extraordinary measures are indeed required in our combined control programs to achieve the Millennium Development Goals for TB/HIV.
